# Relationship difficulties and “technoference” during the COVID-19 pandemic

**DOI:** 10.1177/02654075221093611

**Published:** 2022-11

**Authors:** Giulia Zoppolat, Francesca Righetti, Rhonda N. Balzarini, María Alonso-Ferres, Betul Urganci, David L. Rodrigues, Anik Debrot, Juthatip Wiwattanapantuwong, Christoffer Dharma, Peilian Chi, Johan C. Karremans, Dominik Schoebi, Richard B. Slatcher

**Affiliations:** 1 1190 Vrije Universiteit Amsterdam, The Netherlands; 2 7174 Texas State University, San Marcos, TX, USA; 3 83143 University of Granada, Granada, Spain; 4 5922 Cornell University, Ithaca, NY, United States; 5 56061 Iscte–Instituto Universitário de Lisboa, Lisboa, Portugal; 6 30657 University of Lausanne, Lausanne, Switzerland; 7 26683 Chulalongkorn University, Bangkok, Thailand; 8 7938 University of Toronto, ON, Canada; 9 59193 University of Macau, China; 10 Radboud University Nijmegen, The Netherlands; 11 Université de Fribourg, Fribourg, Switzerland; 12 1355 University of Georgia, Athens, GA, United States

**Keywords:** COVID-19 pandemic, romantic relationships, phubbing, technoference, social media, relationship satisfaction

## Abstract

The COVID-19 pandemic has touched many aspects of people’s lives around the world, including their romantic relationships. While media outlets have reported that the pandemic is difficult for couples, empirical evidence is needed to test these claims and understand *why* this may be. In two highly powered studies (*N* = 3271) using repeated measure and longitudinal approaches, we found that people who experienced COVID-19 related challenges (i.e., lockdown, reduced face-to-face interactions, boredom, or worry) also reported greater self and partner phone use (Study 1) and time spent on social media (Study 2), and subsequently experienced more conflict and less satisfaction in their romantic relationship. The findings provide insight into the struggles people faced in their relationships during the pandemic and suggest that the increase in screen time – a rising phenomenon due to the migration of many parts of life online – may be a challenge for couples.

When the COVID-19 outbreak was declared a global pandemic (Ducharme, 2020), many wondered what the impact of this historic event would be around the world (e.g. Cohut, 2020). This new shared reality would come to affect many aspects of human life, including people’s social relationships. Romantic relationships, in particular, are likely to be dramatically affected by the social isolation and other difficulties driven by the pandemic (Pietromonaco & Overall, 2021; Overall et al., 2021). While it may not be surprising that relationships suffered during this time, given the tight link between environmental stressors and relationship functioning (e.g. Neff & Karney, 2017), research is needed to understand *why* some couples are having a difficult time during this global crisis, particularly given the lasting effects that the pandemic may have worldwide (Holmes et al., 2020). While many factors may play an important role in how relationships are faring, this paper seeks to examine possible drivers and mechanisms behind these initial reports and test how unique aspects related to the COVID-19 pandemic are shaping people’s intimate relationships.

A distinctive characteristic of the pandemic is that, because of social distancing rules encouraged or enforced in communities around the world (United Nations, 2020), millions of people found themselves spending considerably more time online than before the pandemic (GlobalWebIndex, 2020). While technology allows many to continue to connect, work, stay informed, and have fun during periods of lockdown and isolation, it can also interfere with the quality of in-person social interactions (e.g., McDaniel & Coyne, 2016). Did an increase in screen time play a role in how the COVID-19 pandemic influenced people’s romantic relationships?

## Relationships and technology use during COVID-19

The debate as to whether and to what degree the use of technology influences relationships is ongoing (for reviews, see Kushlev & Leitao, 2020 and Sbarra et al., 2019). While technology, such as the use of smartphones, can help people feel connected to others (Pettigrew, 2009) and provide support during stressful times (Holtzman et al., 2017), it can also interfere with relationships (McDaniel & Coyne, 2016) and render social interactions less enjoyable (Aagaard, 2016). Part of the negative association between screen time and relationship quality has been attributed to a phenomenon called *technoference*, whereby the use of technology interferes with in-person social interactions (McDaniel & Coyne, 2014). Technoference can occur through a variety of mediums, but phone use and time spent on social media in particular have been linked to poorer relationship dynamics and outcomes. In the context of romantic relationships, phone use can detract from meaningful in-person connection and take time away from engaging in enjoyable activities with one’s partner (e.g.McDaniel et al., 2018). Partner “phubbing” (i.e., snubbing the other by using one’s phone), a form of technoference, occurs frequently, is felt as problematic by both the phubbed partner (e.g. Lenhart & Duggan, 2014) as well as the one doing the phubbing (Kushlev & Leitao, 2020), and is linked with greater conflict and, ultimately, poorer relationship satisfaction (e.g. McDaniel & Coyne, 2016; McDaniel et al., 2020; Roberts & David, 2016; Wang et al., 2017), both in the short and long term (Halpern & Katz, 2017). Social media use can also interfere with healthy relationship functioning by distracting partners from meaningful interactions (Hand et al., 2013), disrupting communication (Tong & Walther, 2011), and, like phubbing, can cause issues in the relationship (e.g., greater conflict, Lenhart & Duggan, 2014; jealousy, Muise et al., 2009; lower relationship satisfaction and commitment, Quiroz & Mickelson, 2021).

Given that social relationships are central to personal psychological and physical well-being (e.g., Holt-Lunstad et al., 2010), and can be particularly important during stressful times (e.g. Pietromonaco & Collins, 2017), such as during the COVID-19 pandemic (Pietromonaco & Overall, 2021), the worry that technology and screen time negatively affect people’s relationships is legitimate. Indeed, frequent technology use can have cumulative social costs for people’s relationships (Kushlev et al., 2019). Although people use technology for a variety of reasons, particularly to stay connected with others, thwart boredom, and seek information (Stockdale & Coyne, 2020; Whiting & Williams, 2013), these reasons may have become even more important — and thus technoference potentially more present — within the COVID-19 pandemic. In this context, where people were suddenly cut off from their usual social lives, the drive to use technology may have been much stronger than before. For example, people have presumably been using technology more to cope with the worry and distress from the pandemic (Garfin, 2020). A recent study found that both adolescents and adults increased their technology and social media use during the pandemic to connect more with others and gather information, particularly when experiencing anxiety (Drouin et al., 2020). Thus, technology may have served as a replacement tool for the missing in-person social interactions (Drouin et al., 2018), as well as a means to manage worries (Lee & Hawkins, 2016; Juvonen et al., 2021), such as health, social isolation, and financial preoccupations. Moreover, pandemic-related constraints elicited greater feelings of boredom (Boylan et al., 2020), and people who experience boredom engage in phubbing behavior more frequently (Al-Saggaf et al., 2019), with potential consequences for their relationship wellbeing (e.g. McDaniel et al., 2020). Thus, the shift toward technology may have also replaced or distracted people from deeper off-line connections with romantic partners in a time in which a partner’s support is particularly important as a buffer to the outside stressors (Balzarini et al. (in press)). In this way, pandemic-related stressors may have spilled over into people’s most intimate relationships, both directly (Neff & Karney, 2017) and through the increased use of technology (Kushlev & Leitao, 2020).

The present paper seeks to understand whether contextual factors related to the COVID-19 pandemic were related to higher technoference (phone and social media use) and poorer relationship outcomes, adding to the budding and complex body of literature on the influence of technology in relationships as well as to our understanding of the unique context of the COVID-19 pandemic on people’s social lives around the world.

## Research overview

Two studies were conducted to test the link between the COVID-19 pandemic, ‘technoference’ (operationalized as phubbing and social media use)^1^, and relationship outcomes for people in a romantic relationship. The first was a 10-day daily diary study in the Netherlands. By pure coincidence, about half of the diary days were completed before the announcement of COVID-19 lockdown measures in the Netherlands and half were completed during lockdown. This provides valuable data to test whether people experienced their relationships differently in lockdown. Given this opportunity, we tested whether lockdown timing (before vs. during) was related to greater difficulties in relationships and, ultimately, to poorer relationship satisfaction, and whether this was partly explained by their own and their partner’s phubbing behavior. The second study, specifically designed to examine people’s experiences during the COVID-19 pandemic, assessed these effects over time using a longitudinal design with participants from 57 countries. We investigated the specific contextual reasons behind technoference during the pandemic. Specifically, in both cross-sectional and lagged models, we expected that people who experienced greater worry and personal threat related to COVID-19, who engaged in less frequent in-person interactions, and who were more bored, would use social media more and that this would be linked with greater conflict in their relationship and, in turn, with poorer relationship satisfaction. We selected worry, threat, lack of in-person interaction, and boredom as predictors given recent research highlighting these as common pandemic experiences that also have been linked with greater technology use (e.g. Boylan et al., 2020; Drouin et al., 2020; Garfin, 2020), as discussed above. We chose relationship satisfaction as the outcome variable in both studies because it is the most widely used and accepted indicator of relationship quality in relationship science (for a review, see Joel et al., 2020), and chose conflict as its predictor, as conflict in the relationship counts among the most robust predictors of relationship satisfaction (Joel et al., 2020).

## Study 1

### Methods

#### Participants

Participants were 172 individuals in a romantic relationship (mean relationship length was 2.4 years, *SD* = 1.9; range: 4 months - 13 years). Two more participants were excluded from all analyses because they indicated that their data were unreliable. All participants lived in the Netherlands, 40% cohabitated with their partner, and only one had children. Participants were 22 years old on average (*SD* = 3.0; range: 18–33 years), primarily women (75%, 25% men), and identified as heterosexual (80%, 12.8% as bisexual, 5.9% as homosexual, and 1.2% did not identify with the provided options). Participants were recruited by a team of research assistants, on social media, and through a university website. Sample size was determined following guidance for best practices in relationship science (Finkel et al., 2015), as well as financial and practical constraints. Power analyses were conducted in R (version 3.6.3) following approaches recommended by Lane and Hennes (2018) and Kumle et al. (2020) for estimating power for multilevel models, with simulations showing > 80% to detect the smallest effect size of interest for each model path (*b* = .30, .11, & .10 lockdown and phone use, phone use and relationship difficulties, and difficulties and relationship satisfaction, respectively; see Supplemental Material).

### Procedure

Before starting the diary, participants completed an online intake survey on a rolling basis in which they signed informed consent, completed demographics and baseline questionnaires^2^, and were given instructions for the daily diary study, which started on the following Tuesday. The first participants started the diary on March 3rd 2020, and the last started on April 21st. For 10 consecutive days, participants received an email at 8.00 p.m. with a link to the daily questionnaire. The survey was administered through Qualtrics and participants were instructed to complete it before midnight and to do so alone and in a quiet environment. Each day of the diary was coded as occurring either before restrictive COVID measures (coded as 0) or during (coded as 1). While the nationwide lockdown was announced in the Netherlands on March 15th (Darroch, 2020), the suspension of in-person activities (both educational, such as classes and examinations, and social) by Dutch universities started on March 13th (Pieters, 2020).^3^ Given that the participants were Dutch university students, we used the later date to code the “*Lockdown*” variable. Out of the 1732 responses received throughout the diary study, almost half (49.1%) occurred before the universities’ announcements and half during lockdown. Participants were compensated through academic credit or monetarily (receiving up to €40 for the intake survey and when completing at least 80% of the daily diaries). Participants completed 1497 signals (86.5% of the total possible signals), and on average completed 8.7 days (out of 10 possible days, *SD* = 2.15).

### Measures

Given the study employed a repeated measure design, single-item measures were used to reduce fatigue, increase efficiency, and minimize participant attrition (Bolger et al., 2003).

**Technoference.** Every day, participants reported on their *phone use* while in the presence of their partner (1-item; “How much did you use your phone in presence of your partner today?”) and their *partner’s perceived phone use* while in their presence (1-item; “How much did your partner use their phone in your presence today?”), both on a 7-point scale from 1 = *Not much* to 7 = *A lot*.

**Relationship variables.** Every day, participants also rated their *relationship difficulties* (1-item; “How much did you experience problems, difficulties, or struggles in your relationship today?”) on a 7-point scale from 1 = *Not at all* to 7 = *Very much*, and *relationship satisfaction* (1-item; “Today, I feel satisfied with my relationship with my partner”) on a 7-point scale from 1 = *Not at all satisfied* to 7 = *Extremely satisfied*. See Tables 1 and 2 for descriptive statistics and correlations among variables of interest.^4^

**Table 1.** Descriptive statistics in Study 1.

	Before lockdown		During lockdown	
Variables of interest	*M*	*SD*	*M*	*SD*
Self phone use	2.34	1.46	2.93	1.69
Partner phone use	2.58	1.60	3.17	1.74
Relationship difficulties	1.93	1.39	2.25	1.46
Relationship satisfaction	5.90	1.22	5.69	1.32

*Note.* Mean and Standard Deviations of outcome measures, before and during Dutch lockdown. All variables were assessed on a scale of 1–7.

**Table 2.** Correlations among variables of interest in Study 1.

Variables of interest	1	2	3	4
1. Self phone use	.42^a^			
2. Partner phone use	.76**	.38^a^		
3. Relationship difficulties	.10**	.13**	.24^a^	
4. Relationship satisfaction	−.07*	−.07**	−.62**	.39^a^

*Note.* Correlations represent zero-order correlations across all diary days.

*p < .05; **p < .01.

^a^Indicate intra-class correlations.

### Results

#### Analytical strategy

**Figure 1.**
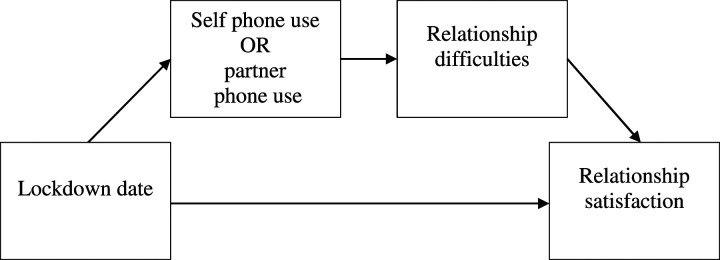
The two serial mediation models from Study 1 of lockdown date (pre-lockdown or during lockdown) on relationship satisfaction through self-reported phone or perceived partner phone use and relationship difficulties.

Given the nested structure of the data (multiple measurements within participants), two-level multilevel analyses were performed (Bolger & Laurenceau, 2013). Intercepts were allowed to randomly vary while slopes were treated as fixed effects. For mediation analyses, continuous predictors were grand-mean-centered to examine between-person effects of the lockdown (Enders & Tofighi, 2007)^5^, allowing to test whether lockdown was related to greater (or lower) self-reported or perceived partner phone use and whether higher (or lower) phone use was associated with higher (or lower) relationship difficulties and relationship satisfaction (see Figure 1). Mediations were tested by first conducting a significance test of parameter estimates for each mediation pathway and then testing the indirect effects (Taylor et al., 2008; Yzerbyt et al., 2018) using the Monte Carlo method for assessing mediation (MCMAM), which is a bootstrapping technique that uses unstandardized estimates to test indirect effects with 95% confidence intervals using 20,000 simulations (Selig & Preacher, 2008). Self phone use and partner phone use were considered separately in each analysis.

#### Key findings

First, we regressed relational indices during the 10-day Diary on lockdown and found, in separate models, a significant association between lockdown and relationship difficulties (*b* = .34, *SE* = .10, 95% CI [.14, .54], *p* = .001) and relationship satisfaction (*b* = −.19, *SE* = .09, 95% CI [-.37, −.002], *p* = .048), with people experiencing poorer relationship quality on days during the lockdown compared to days assessed before lockdown. Next, to test whether self phone use and perceived partner phone use mediated the effects of the lockdown on relationship difficulties, we conducted two separate mediation analyses. In the first step, in separate models (one with self phone use and one with partner phone use), we found a significant association between lockdown and self phone use (*b* = .31, *SE* = .12, 95% CI [.08, .54], *p* = .007) and perceived partner phone use (*b* = .39, *SE* = .12, 95% CI [.15, .63], *p* = .002), with greater phone use reported on days during the pandemic compared to days before the lockdown. In the second step, again in separate analyses, results revealed a significant main effect of self-reported phone use (*b* = .09, *SE* = .03, 95% CI [.03, .14], *p* = .001) and perceived partner phone use (*b* = .09, *SE* = .02, 95% CI [.04, .13], *p* < .001) on relationship difficulties controlling for lockdown. Indirect effects did not contain zero, indicating that self and partner phone use were, in separate models, significant mediators between lockdown and relationship difficulties. Results are displayed in Table 3.

**Table 3.** Results of the multilevel mediation analysis in Study 1 on the effect of the lockdown on relational outcomes through greater self and partner phone use and relationship difficulties.

Predictor and effect	Main model			
	*b*	*SE*	95% CI	*p*
	Outcome: Relationship difficulties			
Self phone use^a^	.09	.03	[.03, .14]	.001
Pandemic lockdown				
Total effect	.34	.10	[.14, .54]	.001
Direct effect	.31	.10	[.10, .51]	.003
Indirect effect	.03		[.01, .06]	
	Outcome: Relationship difficulties			
Partner phone use^a^	.09	.02	[.04, .13]	<.001
Pandemic lockdown				
Total effect	.34	.10	[.14, .54]	.001
Direct effect	.32	.12	[.10, .50]	.003
Indirect effect	.03		[.01, .07]	
	Outcome: Relationship satisfaction			
Relationship difficulties^a^	−.47	.02	[−.51, −.44]	<.001
Self phone use				
Total effect	−.05	.02	[−.09, −.01]	.03
Direct effect	−.002	.02	[−.04, .03]	.90
Indirect effect	−.04		[−.07, −.02]	
	Outcome: Relationship satisfaction			
Relationship difficulties^a^	−.48	.02	[−.51, −.44]	<.001
Partner phone use				
Total effect	−.04	.02	[−.7, .003]	.068
Direct effect	.01	.02	[−.03, .04]	.677
Indirect effect	−.04		[-.07, −.02]	

^a^indicates *b* pathways in the mediation models and were estimated controlling for *a* pathways of the predictor variables.

Finally, we tested whether relationship difficulties mediated the link between phone use and relationship satisfaction. In separate models, we found a significant association between self phone use (*b* = .09, *SE* = .03, 95% CI [.04, .14], *p* < .001) and perceived partner phone use (*b* = .09, *SE* = .02, 95% CI [.05, .14], *p* < .001) on relationship difficulties. Next, again in separate analyses, results revealed a significant main association between relationship difficulties and relationship satisfaction, when controlling for either self phone use or perceived partner phone use. Indirect effects did not contain zero for both models (self phone use and perceived partner phone use), indicating that phone use was related to greater difficulties in the relationship and, subsequently, with poorer relationship satisfaction.^6^ Again, results are displayed in Table 3.

### Discussion

The present study offers a unique opportunity to compare pre- and during-lockdown relationship quality, and provides initial, yet compelling, evidence that relationships were generally worse on days during the pandemic compared to those assessed before lockdown policies, and that an increase in phone use partially played a role. This is in line with the robust body of literature that highlights the detrimental effect of outside stressors on relationship functioning (e.g., Neff & Karney, 2017). Notably, the total effect of the pandemic lockdown on relationship satisfaction was small albeit significant, highlighting instead the role that phone use, and the relationship challenges reported thereof, played when it comes to relationship quality before versus during lockdown. Interestingly, both own phone use and partner phone use were related to poorer relationship outcomes, suggesting that both being phubbed and doing the phubbing are detrimental for relationships, which is in line with other research showing the negative effects of both on wellbeing (Ergün et al., 2020). However, given that the study had not originally been planned to specifically examine relationships during the COVID-19 pandemic, the analyses were exploratory. To gain greater generalizability, we conducted a second study — with a larger and more diverse sample — specifically designed to examine relational experiences during the COVID-19 pandemic. In this study, we again tested the association between screen time and relationship outcomes, as well as the possible drivers of these associations, to examine the contextual factors that may have played a role.

## Study 2

### Methods

#### Participants

Participants were drawn from six time points (measured every 2 weeks, covering 3 months) of an ongoing longitudinal study on the effects of the COVID-19 pandemic on social relationships (see OSF). Participants had to be at least 18 years old and were recruited through word of mouth, social media, and the project’s website. The study was launched on March 27th, shortly after the global pandemic was declared by the World Health Organization (Ducharme, 2020). The study was first available in English and then translated into 10 other languages (Spanish, Turkish, Thai, Chinese, Dutch, French, German, Italian, Indonesia, and Portuguese) through a process of back-translation, a technique frequently used in cross-cultural research to minimize discrepancies (Colina et al., 2017). In total, 5571 people from 57 different countries participated in the study (country-level demographic information can be found in the Supplemental Material). For the present investigation, we selected participants who were in a romantic relationship (*N* = 3120); participants who indicated poor quality of attention to the survey were excluded (1-item; “How much attention did you pay to this questionnaire while you were completing it?” on a 4-point scale from *No attention* to *Very Close Attention*). The final sample size was 3099 participants. Participants were on average 33 years old (*SD* = 12; range:18–82 years), mostly women (81.4%, 16.7% men, and 1.3% did not identify with the provided options), had a university degree (72.8%), identified as heterosexual (82.3%, 11.7% as bisexual, 3.7% as lesbian/gay, and 2.3% did not identify with the provided options), and most lived with their partner (56.7%). Most participants reported living in the United States (30%) or Spain (23.4.7%), followed by Turkey (7.8%), Switzerland (6.7%), and Portugal (5.0%) (for the full country list and percentages see Supplemental Material). Power analyses showed > 80% power to detect the smallest effects of interest for all pathways (*b =* .05, .10, .04 for predictors and social media see, social media use and difficulties, and difficulties and satisfaction, respectively; see Supplemental Material).

### Measures

Given the longitudinal nature of the study, single-item measures were used to maximize participant retention and minimize fatigue (Bolger et al., 2003).

**Pandemic factors.** Participants rated various pandemic related stressors, including their *perception of pandemic seriousness* (1-item; “The current situation due to the COVID-19 virus is very serious”) and their *perception of personal threat* (1-item; “The current situation due to the COVID-19 virus feels personally threatening: financially, emotionally, or physically”), both on a 5-point scale from *Not at all* to *Completely,* their *time spent face-to-face with family, friends, and colleagues* (3-items; “Over the past 2 weeks, how much time have you spent with family/friends/colleagues face-to-face?”) on a 4-point scale from *Never* to *Every day,* and how *bored* they felt over the last 2 weeks on a 5-point scale from *Very slightly or not at all* to *Extremely*.

**Social media use.** Participants then rated their self-reported *social media use* (1-item; “Over the past 2 weeks, on average, roughly how many hours have you spent each day on social media?”) on a scale of *0 hours, up to 1 hour*, *1–3 hours*, *3–7 hours*, *8 or more hours.*

**Relationship variables.** Participants reported their *relational conflict* (1-item adapted from Braiker & Kelley, 1979; “How often did you and your partner argue with each other over the last 2 weeks?”) measured on a 7-point scale from *Not very often* to *Very often*, as well as their *relationship satisfaction* (1-item from the Perceived Relationships Quality Component Scale, Fletcher et al., 2000; “Thinking about your feelings over the last 2 weeks, how satisfied are you with your partner?”) measured on a 7-point scale from *Not at all* to *Extremely*. See Table 4 for descriptive statistics.

**Table 4.** Means, standard deviations, and correlations among variables of interest in Study 2.

Variables of interest	M	SD	1	2	3	4	5	6	7	8	9
1. Pandemic seriousness	4.16	.91	.59^a^								
2. Pandemic as personal threat	3.45	1.14	.44**	.61^a^							
3. Boredom	2.67	1.28	.05**	.19**	.66^a^						
4. Time spent face-to-face with friends	1.54	.75	−.17**	−.15**	−.05**	.50^a^					
5. Time spent face-to-face with family	2.75	1.29	.01	.05**	−.01	−.06**	.65^a^				
6. Time spent face-to-face with colleagues	1.34	.72	−.14**	−.10**	−.07**	.26**	−.03*	.69^a^			
7. Social media use	3.23	.88	.16**	.21**	.27**	−.06**	.02	−.06**	.67^a^		
8. Relationship conflict	2.29	1.6	.03*	.10**	.17**	−.01	.01	.001	.08**	.59^a^	
9. Relationship satisfaction	5.87	1.30	.06**	−.03**	−.10**	.01	.01	.01	−.02	−.42**	.71^a^

*Note.* All variables were assessed on a scale of 1–5, except for relationship conflict and satisfaction, which were assessed on a scale of 1–7. Correlations represent zero-order correlations across all measurement time points.

*p < .05; **p < .01.

^a^Indicate intra-class correlations.

### Results

#### Analytical strategy

**Figure 2.**
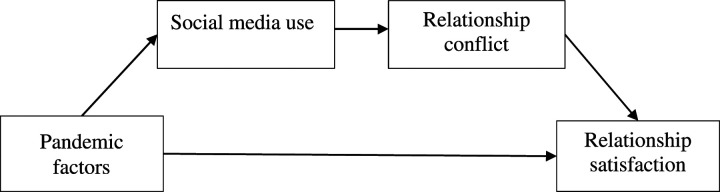
The serial mediation models from Study 2 of pandemic factors (perception of pandemic seriousness, perception of threat, time spent with friends, family, and colleagues face-to-face, and boredom) on relationship satisfaction through self-reported social media use and relationship conflict.

Given the multiple measurements per participant, two-level multilevel analyses with random intercepts and fixed slopes were performed to account for the nested nature of the data (Bolger & Laurenceau, 2013). Mediations were tested as in Study 1, by first conducting parameter estimates for each mediation pathway and then testing the indirect effects using MCMAM. This allowed us to test whether participants who experienced greater pandemic stressors (each considered separately) also reported greater social media use and whether this, in turn, was related to greater conflict and poorer relationship satisfaction (see Figure 2*).* We first conducted the analyses cross-sectionally, and then examined these effects over time using time-lagged multilevel analyses across the six data time points to test whether our predictors would be related to changes in the outcome variables at the subsequent time points (Selig & Little, 2012). Specifically, we tested whether earlier pandemic factors predicted changes in later relationship quality (controlling for earlier relationship quality) and whether this change was mediated by social media use. By considering earlier relationship quality in the models, it is possible to determine whether it is indeed the predictor variables (pandemic factors and social media use) to drive the effects on relationship outcomes above and beyond earlier levels of relationship quality.

#### Key findings

As expected, in separate models (i.e. with each pandemic factor considered separately), social media use was significantly associated with perception of pandemic seriousness (*b* = .16, *SE* = .01, 95% CI [.14, .19], *p* < .001), perception of personal threat (*b* = .13, *SE* = .01, 95% CI [.11, .14], *p* < .001), time spent with friends face-to-face (*b* = −.08, *SE* =. 01, 95% CI [-.11, −.06], *p* < .001), time spent with family face-to-face (*b* = .02, *SE* =. 01, 95% CI [.007, .04], *p* < .001), time spent with colleagues face-to-face (*b* = −.08, *SE* =. 02, 95% CI [-.11, −.05], *p* < .001), boredom (*b* = .14, *SE* = .01, 95% CI [.12, .15], *p* < .001). Next, in separate models, results revealed a significant positive association between social media use and relationship conflict controlling for each pandemic factor (except boredom). Indirect effects’ CI did not contain zero, indicating that social media use was a significant mediator between pandemic factors and relationship conflict in all models (except boredom). Finally, to test the full serial mediation, we tested whether social media use was related to relationship satisfaction through the mediating role of conflict. First, we regressed relationship conflict on social media use (*b* = .09, *SE* = .03, 95% CI [.05, .14], *p* < .001), and second, regressed relationship satisfaction on conflict controlling for social media use, and found a significant main effect. Again, the indirect effect’s CI did not contain zero, indicating that social media use was related to lower relationship satisfaction through greater conflict with the partner.^7^ See Table 5 for all statistics.

**Table 5.** Results of the cross-sectional multilevel mediation analysis in Study 2 on the effect of pandemic seriousness, pandemic threat, and time spent with friends, family, or colleagues, and boredom on relational outcomes through social media use and relationship conflict.

Predictor and effect	Main model			
	*b*	*SE*	95% CI	*p*
	Outcome: Relationship conflict			
Social media use^a^	.09	.02	[.04, .14]	<.001
Pandemic seriousness				
Total effect	.03	.02	[−.01, .08]	.156
Direct effect	.02	.02	[−.03, .06]	.448
Indirect effect	.01		[.01, .02]	
	Outcome: Relationship conflict			
Social media use^a^	.08	.02	[.03, .12]	.002
Pandemic threat				
Total effect	.09	.02	[.06, .14]	<.001
Direct effect	.08	.02	[.04, .12]	<.001
Indirect effect	.01		[.003, .02]	
	Outcome: Relationship conflict			
Social media use^a^	.10	.02	[.05, .14]	<.001
Time spent with friends				
Total effect	.02	.03	[−.04, .06]	.659
Direct effect	.02	.03	[−.03, .07]	.458
Indirect effect	−.01		[−.01, −.004]	
	Outcome: Relationship conflict			
Social media use^a^	.09	.02	[.05, .14]	<.001
Time spent with family				
Total effect	−.01	.02	[−.04, .03]	.737
Direct effect	−.01	.02	[−.04, .03]	.644
Indirect effect	.002		[.001, .004]	
	Outcome: Relationship conflict			
Social media use^a^	.10	.02	[.05, .14]	<.001
Time spent with colleagues				
Total effect	−.01	.03	[−.07, .06]	.856
Direct effect	.002	.03	[−.06, .06]	.952
Indirect effect	−.01		[−.01, −.003]	
	Outcome: Relationship conflict			
Social media use^a^	.05	.03	[−.002, .09]	.062
Boredom				
Total effect	.17	.02	[.13, .20]	<.001
Direct effect	.16	.02	[.13, .19]	<.001
Indirect effect	.01		[−.0003, .01]	
	Outcome: Relationship satisfaction			
Conflict^a^	−.27	.01	[−.29, −.29]	<.001
Social media use				
Total effect	.01	.02	[−.03, .04]	.758
Direct effect	.03	.02	[−.007, .06]	.128
Indirect effect	.02		[−.03, −.01]	

^a^indicates *b* pathways in the mediation models and were estimated controlling for *a* pathways of the predictor variables.

Next, six-wave time-lagged regression analyses were conducted to test the hypotheses over time, first testing whether earlier pandemic factors (at *t*) and social media use were related to changes in conflict at a subsequent time point (at *t + 1*), controlling for conflict at the previous time point (at *t*). See Table 6 for all statistics. In separate models, results revealed a significant main effect of social media use on later relationship conflict controlling for earlier pandemic factors. Results also revealed a significant main effect of conflict on relationship satisfaction at a subsequent time point controlling for earlier relationship satisfaction, earlier conflict, and social media use. Lastly, the indirect effects’ CI did not contain zero, indicating that social media use was a significant mediator between earlier pandemic stressors and relationship outcomes at a later time point. Finally, to account for the important differences in COVID-19 pandemic policies across countries and regions (Cohut, 2020), we tested whether our effects held when controlling for local social distancing policies (overall, social distancing had been encouraged in 33% of cases, ordered in 30.6%, enforced in 32.5%, and no social distancing policies had been reported in 3.9% of cases). The pattern of results remained the same (see Supplemental Material for details).

**Table 6.** Results of the lagged mediation analysis in Study 2 on the effect of pandemic seriousness, pandemic threat, and time spent with friends, family, or colleagues, and boredom (at *t*), on relational outcomes at a later time point (*t + 1*) through earlier social media use and conflict.

Predictor and effect	Main lagged model			
	*b*	*SE*	95% CI	*p*
	Outcome: Later relationship conflict			
Social media use	.09	.03	[.03, .14]	.001
Pandemic seriousness				
Total effect	.02	.03	[−.03, .07]	.405
Direct effect	.01	.03	[−.04, .06]	.77
Indirect effect	.01		[.01, .02]	
	Outcome: Later relationship conflict			
Social media use	.07	.03	[.02, .12]	.008
Pandemic threat				
Total effect	.07	.02	[.03, .11]	.001
Direct effect	.06	.02	[.02, .10]	.007
Indirect effect	.01		[.002, .02]	
	Outcome: Later relationship conflict			
Social media use	.09	.03	[.04, .14]	.001
Time spent with friends				
Total effect	−.01	.03	[−.06, .06]	.879
Direct effect	.01	.03	[−.06, .07]	.887
Indirect effect	−.01		[−.01, −.002]	
	Outcome: Later relationship conflict			
Social media use	.09	.03	[.04, .14]	.001
Time spent with family				
Total effect	−.01	.0	[−.04, .03]	.77
Direct effect	−.01	.02	[−.04, .03]	.773
Indirect effect	.002		[.0004, .004]	
	Outcome: Later relationship conflict			
Social media use	.09	.03	[.04, .14]	.001
Time spent with colleagues				
Total effect	.03	.03	[−.03, .01]	.317
Direct effect	.04	.03	[−.02, .11]	.207
Indirect effect	−.01		[−.01, −.003]	
	Outcome: Later relationship conflict			
Social media use	.07	.03	[.02, .12]	.011
Boredom				
Total effect	.06	.02	[.03, .10]	<.001
Direct effect	.05	.02	[.02, .01]	.005
Indirect effect	.01		[.01, .001]	
	Outcome: Later relationship satisfaction			
Relationship conflict	−.23	.01	[−.25, −.20]	<.001
Social media				
Total effect	−.05	.02	[−.08, −.02]	.008
Direct effect	−.03	.02	[−.06, .01]	.128
Indirect effect	−.02		[−.03, −.01]	

^a^indicates *b* pathways in the mediation models and were estimated controlling for path *a* of the predictor variable as well as relationship variables at an earlier time point.

### Discussion

Overall, results from Study 2 indicate that people who reported greater pandemic challenges also reported using social media more and experienced greater conflict in their relationship and poorer relationship satisfaction, both cross-sectionally and over time, above and beyond their original levels of relationship quality (in the lagged analysis). Total effects were not always significant, suggesting perhaps that not all pandemic stressors were equally disruptive to relationships. Rather, pandemic stressors were consistently related to greater social media use which, in turn, was consistently related to poorer relationship outcomes. Curiously, and contrary to our hypotheses, while time spent with friends and family face-to-face was related to less time on social media as predicted, time spent face-to-face with family was linked with *more* time on social media. This may be a symptom of the same mechanism hurting romantic relationships in this context, with technology intruding on people’s time with their family as well as with their partner (McDaniel, 2015). Overall, these results build off those from Study 1, provide longitudinal evidence, and test the hypotheses in a larger and more diverse sample.

## General discussion

In two studies, the present investigation finds that romantic relationships suffered during the pandemic, with greater self and partner phone use (Study 1) and time spent on social media (Study 2) mediating the link between COVID-19 related factors and poorer relationship outcomes. Study 1 offers a unique dataset to compare relationship perceptions before and during lockdown, finding that perceptions of relationship quality differed, with relationships assessed on days before lockdown measures were put in place faring better than those assessed during lockdown days. Study 2 used a larger and more diverse sample to examine why the pandemic was related to worse relationship outcomes over time, suggesting that people who experienced the pandemic as more serious in general as well as personally threatening, those who engaged in less face-to-face interactions with friends and colleagues, and who experienced boredom, turned to social media more, subsequently reporting lower romantic relationship quality.

These findings are in line with early media reports speculating about how the pandemic would affect relationships (Prasso, 2020), as well as the reports of the drastic changes to online behavior (GlobalWebIndex, 2020). On a global scale, people were faced with existentially threatening situations, such as loss of work and income, new health worries, and fewer opportunities for in-person social interactions (Brooks et al., 2020). These and other environmental factors spill over into people’s intimate relationships (Neff & Karney, 2017), and preliminary scientific evidence has found that people who experienced greater stress, financial strain, and loneliness also experienced lower relationship quality during the pandemic (Balzarini et al., in press). Our work suggests that challenges related to the COVID-19 pandemic, while not always directly linked with poorer relationship quality, spilled over into people’s relationships partially through the greater use of technology. While technology has become part of everyday life, the extent to which many aspects of people’s lives revolved around technology on a global scale is unique to the present crisis (GlobalWebIndex, 2020), presenting an important opportunity to contribute data to the ongoing debate on how technology affects relationships. While technology and social media have not always been found to affect wellbeing, particularly during “normal” times (Kushlev & Leitao, 2020), the pandemic-related factors suggest a more complex story, in line with recent calls for researchers to consider the context when investigating the link between technology and relationships (Kushlev & Leitao, 2020; Sbarra et al., 2019). Indeed, although greater use of technology may have allowed people to stay connected to their outside worlds, such as by enabling remote work (Brooks et al., 2020) and connecting to friends (Juvonen et al., 2021), it also meant that people were likely less present in their in-person relationship. The evidence for this is particularly strong in Study 1, where people reported specifically on their own or partner’s phone use *while in the presence of the other.* Thus, in line with the displacement and interference hypothesis of technoference (Kushlev & Leitao, 2020), phone use and social media can be harmful when they take away from quality time with a partner and interfere with their relationship (e.g. McDaniel et al., 2018). While we were not able to directly test this, it is possible that by spending more time on their phones and social media, people may have lost opportunities to be present with their partner, to connect and provide support, which are important aspects for healthy relationship development (e.g. Gordon & Chen, 2016), particularly during times of stress (Balzarini et al., in press).

The present work builds off the burgeoning literature on the role of technology in relationships by going beyond one-time assessments and provides much needed well-powered repeated measure and longitudinal studies to test when technology can be an issue for relationships (Sbarra et al., 2019). While some recent work has examined technoference over time and in daily life (e.g. McDaniel & Drouin, 2019), highlighting its negative influence on relationship quality, little is known about its antecedents (and, to our knowledge, if it has done so, it has mainly used one-time assessments; e.g. Drouin & McDaniel, 2021). Our work takes the wider context into consideration, highlighting how outside factors — those associated with the COVID-19 pandemic — are related to greater technology use. Our results corroborate research showing that people spend more time using technology when experiencing greater stressors (e.g. feeling worried, when feeling alone, or bored; Al-Saggaf et al., 2019; Drouin et al., 2018; Juvonen et al., 2021) and is in line with the stress-spillover hypotheses (Neff & Karney, 2017), whereby external stressors can spillover into romantic relationships, negatively influencing relationship quality. The present work suggests that one way in which these stressors do so is through greater technology use.

It is worthy to note that our exploratory analyses revealed some mixed results with regards to the level at which the association between technology and relationship quality occurred. Both within- and between-person associations were found in Study 1, such that people who used their phone or perceived their partner to use their phone more than others, as well as those who did so or perceived their partners to do so more than their own usual, reported greater relationship difficulties and worse relationship satisfaction. However, in Study 2, while there were both within- and between-person associations between pandemic stressors and greater social media use, only between-person associations were found for the link between social media use and relational conflicts, such that people who used social media more than others (rather than people who used social media more than their own usual) reported worse relationship quality. This is interesting given the mixed evidence in the literature as to whether and to what degree the use of technology influences relationships (for reviews, see Kushlev & Leitao, 2020 and Sbarra et al., 2019). While our findings suggest that the link between technology use and relationship conflict and satisfaction may more consistently be a between- rather than within-person phenomenon, given the mixed findings in the present study as well as in the general literature (McDaniel et al., 2020), our work is not conclusive on this point. We thus join the call for researchers to continue investigating these associations at both levels to best disentangle when and for whom technology may be nefarious for relationships and when it may not.

We recognize that many other factors are likely to negatively impact romantic relationships during the COVID-19 pandemic, and research will undoubtedly continue to investigate them. The present investigation provides evidence for one possible reason for changes in relationship satisfaction, and offers unique longitudinal evidence of the relationship between pandemic factors, technoference, and relationship outcomes, but is, of course, not exhaustive. A limitation is that the studies measured *perceived* phone and social media use, rather than capturing technology use through objective measures. This is important given that people are not always good at estimating the time they spend online (Kushlev & Leitao, 2020). However, self-reports are a useful measure for the present research question, given the key role that personal perceptions play in relationship outcomes (e.g. Joel et al., 2020). Given that official reports confirmed an increase in technology use around the world (GlobalWebIndex, 2020), we encourage further investigation of the role that technology played in relationships during the COVID-19 pandemic. Specifically, a fruitful avenue for future research would be to compare perceptions of technology use with objective measures that can collect precise assessments of actual use. Although research has already shown that self-report estimates are not strongly related to objective scores of technology use and have thus called for caution when making inferences about the links between self-reported technology use and well-being (Ellis, 2019), the importance of perceptions in romantic relationships in determining relationship outcomes above more objective measures (e.g., Joel et al., 2020) warrants greater attention to the comparison between the perceived and objective role that technology plays in this context. This type of examination could distinguish whether the effects of technology use on relationships is more a matter of perception or objective reality. Furthermore, while our study collected data from individuals in romantic relationships, it would be useful to collect data from both partners and examine the interplay between perception and reality when it comes to people’s own and partner assessments, examining the accuracy and bias of each.

Another limitation of the current study is that, while we conceptualize technoference as occurring through phone (in Study 1) and social media (Study 2), the measure used for social media use in the second study did not specifically assess people’s social media use *while in the presence of their partner*. However, we still observe that people who spent more time on social media experienced greater conflict and poorer relationship satisfaction, suggesting that technology use interfered with people’s relationships, lowering their quality. Finally, not all potentially relevant demographic information was collected (e.g. class, disability status). We are thus not able to generalize our findings beyond the current reported demographics. This is important as technology use can differ among groups (e.g. socio-economic background, Hsu et al., 2015; disability, Dobransky & Hargittai, 2021). Future research should be mindful of this to better investigate the link between external stressors, technology use and relationship quality and how this may or may not differ based on certain meaningful demographic characteristics.

Overall, the present work offers new and unique insight into the world of romantic relationships during the COVID-19 pandemic, showing some of the first empirical evidence of a decline in relationship quality and an increase in technology use as a possible reason for this effect.

## Supplemental material

Supplemental material - Relationship difficulties and “technoference” during the COVID-19 pandemic Click here for additional data file. Supplemental Material for Relationship difficulties and “technoference” during the COVID-19 pandemic by Giulia Zoppolat, Francesca Righetti, Rhonda Balzarini, María Alonso-Ferres, Betul Urganci, David L. Rodrigues, Anik Debrot, Juthatip Wiwattanapantuwong, Christoffer Dharma, Peilian Chi, Johan Karremans, Dominik Schoebi1, and Richard B. Slatcher in Journal of Social and Personal Relationships

## Notes

1. Study 1 only measured self and partner phone use and Study 2 only measured social media use. Because technoference occurs through both these mediums, we adopted both operationalizations as indexes of technoference.

2. This study was part of a larger study with people in romantic relationships. Only variables relevant to the current research question are reported. Full list of measures is available on the OSF.

3. Measures included working from home, ban on large gathering, and general social contact restrictions, including the closure of public spaces such as museums, concerts, theatres, sport clubs and events. A 395-euro fine was established for violators.

4. Participants who completed the intake session before lockdown were a bit younger (*M*_age_=21.26, *SD* = 2.71) than those who completed it during lockdown (*M*_age_=23.62, *SD* = 2.84). However, they did not differ in relationship length (*p* = .203), and, among those who lived with their partner, did not differ in how long they had lived together (*p* = .399). They also did not differ on attachment anxiety (*p* = .698) or avoidance (*p* =.336), which are strong predictors of how people manage relationship stress (e.g., Li & Chan, 2012).

5. Because the study was not originally planned to capture experiences during the pandemic, diary days were completed by some participants completely before the restrictive measures began (*N* = 64), by some completely during (*N* = 68), and others completed some days before and some days during (*N* = 42). Thus, estimating within-person variations would not be advisable, given the reduced power to estimate these effects.

6. We also conducted multilevel analyses in which we examined the associations between perceived phone use and relationship outcomes at both a within and between-person level. Results revealed that on days in which participants perceived greater phone use (their own or their partner’s), they also experienced poorer relationship outcomes, consistent with the between-person effects. Full results are reported in the Supplemental Material.

7. We conducted additional analyses including both the within-person centered predictors as well as their aggregates across all six waves in order to disentangle the within and the between effects. We found a significant between- and within-person association between pandemic factors and social media use, and a significant association between social media use and conflict with the partner, but only at the between person level. Full results are reported in the Supplemental Analysis.
