# Supplementary material for: Relationship difficulties and “technoference” during the COVID-19 pandemic
Source: J Soc Pers Relat. 2022 Nov;39(11):3204–27. doi: 10.1177/02654075221093611 (PMC9630926; doi:10.1177/02654075221093611)
Supplement: Supplemental material - Relationship difficulties and “technoference” during the COVID-19 pandemic [file sj-pdf-1-spr-10.1177_02654075221093611.pdf]

Supplemental Material

Relationship difficulties and "technoference" during the COVID-19 pandemic

Table of Content

Study 1

1) **Analysis: within and between person analyses**

Study 2

2) **Analysis: Controlling for local social distance policy**
3) **Analysis: within and between person analyses**
4) **Country demographics**

Study 1 & 2

5) **Power analyses**

Study 1

1) Analysis: within and between person analyses

We conducted additional exploratory analyses within-person centering the phone use variables to examine within-person variation across diary days and included their aggregate (i.e. each person's average across all diary days) in order to disentangle the within- and between-person differences (Bolger & Laurenceau, 2013; Zhang et al., 2009) between perceived phone use and relationship outcomes.

First, in separate models, we regressed relationship difficulties on own and partner phone use, and found a significant association at a within-person level (b = .10, SE = .03, 95% CI [.05, .16], p < .001) but not between-person (b = .06, SE = .06, 95% CI [-.05, .17], p = .283) for own phone use. The association was significant within-person (b = .09, SE = .03, 95% CI [.04, .14], p = .001) and between-person (b = .12, SE = .06, 95% CI [.01, .24], p = .039) for partner phone use. Next, in separate models, we regressed relationship satisfaction on relationship difficulties, controlling for either self or partner phone use. Results revealed a significant within- and between-person association between relationship difficulties and relationship satisfaction for both own phone use and perceived partner phone use. Indirect effects for within person models did not include zero, indicating that relationship difficulties mediated, in separate models, the negative relationship between own phone use, perceived partner phone use and relationship satisfaction. These analyses indicate that on days in which participants perceived greater phone use (their own or their partner's), they also experienced poorer relationship outcomes, consistent with the original between-person effects. Full results can be seen in Table 1.

Table 1: Between-person and within-person results (regressed concurrently) of the multilevel mediation analysis in Study 1 on the effect of phone use on relational satisfaction through relationship difficulties. * indicates *b* pathways in the mediation models and were estimated controlling for *a* pathways of the predictor variables.

	Between-person				Within-person			
Predictor and effect	*b*	*SE*	95% CI	*p*	*b*	*SE*	95%	*p*
				Outcome: Relationship satisfaction				
Relationship difficulties*	-.78	.06	[-.89, -.67]	<.001	-.44	.02	[-.48, -.41]	<.001
Own phone use								
Total effect	-.05	.06	[-.16, .07]	.425	-.05	.02	[-.09, -.001]	.044
Direct effect	-.002	.04	[-.08, .08]	.951	-.002	.02	[-.04, .04]	<.937
Indirect effect	-.05		[-.13, .04]		-.05		[-.07, -.02]	
				Outcome: Relationship satisfaction				
Relationship difficulties*	-.78	.06	[-.89, -.68]	<.001	-.45	.02	[-.48, -.41]	<.001
Partner phone use								
Total effect	-.06	.06	[-.18, .05]	.266	-.03	.02	[-.07, .01]	.123

Direct effect	.02	.04	[-.06, .10]	.578	.01	.02	[-.03, .04]	.676
Indirect effect	-.09		[-.02, -.02]		-.04		[-.07, -.01]	

Study 2

2) Analyses in Study 2 controlling for local social-distancing policy

Given the wide variety of COVID-19 pandemic related policies, and their potential influence on people's experiences during the pandemic, using the same analytical design to test for mediation, we conducted additional analyses controlling for local social distancing policies, in both the concurrent and lagged models. We created a new "policy" variable related to the social distancing regulations in place in the previous two weeks (0 = no social distancing regulations, 1 = social distancing has been encouraged, 2= social distancing has been ordered, 3 = social distancing has been enforced by the police). We chose to control for social distancing policy as it is an index of how each location has handled the pandemic. Overall, in 33% of cases social distancing had been encouraged, in in 30.6% ordered, in 32.5% enforced, and in 3.9% of cases no social distancing policies had been reported. Controlling for this variable, the pattern of results remains the same, indicating that the personal experiences of the pandemic and their relationship with social media use and changes in relationship outcomes go above and beyond the actual policy measures.

First, in separate models we still find a significant association between pandemic seriousness (b = .15, SE = .01, 95% CI [.13, .18], p < .001), pandemic threat (b = .12, SE = .01, 95% CI [.10, .14], p < .001), time spent with friends (b = -.07, SE = .01, 95% CI [-.10, -.05], p < .001), time spent with family (b = .02, SE = .01, 95% CI [.01, .04], p = .008), time spent with colleagues (b = -.06, SE = .02, 95% CI [-.09, -.03], p < .001), boredom (b = .13, SE = .01, 95% CI [.12, .15], p < .001) and social media use, controlling for local policy. Second, social media was related to poorer relationship outcomes controlling for pandemic factors and local policy (and earlier relationship outcomes in the lagged models). The full tables of results are below.

Table 2: Results of the mediation analysis in Study 2 on the effect of pandemic seriousness, pandemic threat, and time spent with friends, family, or colleagues, and boredom, on relational outcomes through earlier social media use, controlling for local policy. Social media use coefficients are path *b* in the mediation models, and were estimated controlling for path *a* of the predictor variable as well as local policy.

	Main model			
Predictor and effect	*b*	*SE*	95% CI	*p*
	Outcome: relationship conflict			
Social media use	.09	.02	[.04, .14]	<.001
Pandemic seriousness				
Total effect	.03	.02	[-.02, .07]	.234
Direct effect	.01	.02	[-.03, .06]	.557
Indirect effect	.01		[.01, .02]	
	Outcome: relationship conflict			
Social media use	.08	.02	[.03, .12]	.002

Pandemic threat				
Total effect	.08	.02	[.05, .12]	<.001
Direct effect	.08	.02	[.04, .11]	<.001
Indirect effect	.01		[.003, .015]	
		Outcome: relationship conflict		
Social media use	.09	.02	[.05, .14]	<.001
Time spent with friends				
Total effect	.02	.03	[-.03, .07]	.499
Direct effect	.03	.03	[-.03, .09]	.353
Indirect effect	-.01		[-.01, -.003]	
		Outcome: relationship conflict		
Social media use	.09	.02	[.04, .14]	<.001
Time spent with family				
Total effect	-.01	.02	[-.04, .03]	.707
Direct effect	-.008	.02	[-.04, .02]	.623
Indirect effect	.002		[.0004, .004]	
		Outcome: relationship conflict		
Social media use	.09	.02	[.04, .14]	<.001
Time spent with colleagues				
Total effect	.001	.03	[-.06, .06]	.982
Direct effect	.01	.03	[-.06, .06]	.825
Indirect effect	-.006		[-.01, -.002]	
		Outcome: relationship conflict		
Social media use	.04	.02	[-.004, .09]	.071
Boredom				
Total effect	.16	.02	[.13, .20]	<.001
Direct effect	.16	.02	[.13, .19]	<.001
Indirect effect	.006		[-.004, .01]	
		Outcome: relationship satisfaction		
Relationship conflict	-.27	.008	[-.29, -.26]	<.001
Social Media use				
Total effect	.004	.02	[-.03, .04]	.819
Direct effect	.02	.02	[-.009, .06]	.163
Indirect effect	.02		[-.04, -.01]	

Table 3: Results of the lagged mediation analysis in Study 2 on the effect of pandemic seriousness, pandemic threat, and time spent with friends, family, or colleagues, and boredom, on relational outcomes at a later time point through earlier social media use and conflict, controlling for local policy. * indicates *b* pathways in the mediation models and were estimated controlling for path *a* of the predictor variable as well as relationship variables and local policy at an earlier time point.

	Main lagged model			
Predictor and effect	*b*	*SE*	95% CI	*p*

	Outcome: Later relationship conflict			
Social media use	.08	.03	[.03, .13]	.002
Pandemic seriousness				
Total effect	.02	.03	[-.03, .07]	.496
Direct effect	.01	.03	[-.04, .05]	.861
Indirect effect	.01		[.01, .02]	
	Outcome: Later relationship conflict			
Social media use	.07	.03	[.02, .12]	.011
Pandemic threat				
Total effect	.07	.02	[.03, .10]	.001
Direct effect	.05	.02	[.01, .09]	.009
Indirect effect	.01		[.002, .02]	
	Outcome: Later relationship conflict			
Social media use	.08	.03	[.03, .13]	.001
Time spent with friends				
Total effect	-.0003	.03	[-.06, .06]	.993
Direct effect	.008	.03	[-.05, .07]	.797
Indirect effect	-.01		[-.01, -.002]	
	Outcome: Later relationship conflict			
Social media use	.08	.03	[.03, .13]	.001
Time spent with family				
Total effect	-.004	.02	[-.04, .03]	.800
Direct effect	-.004	.02	[-.04, .03]	.799
Indirect effect	.002		[.0003, .004]	
	Outcome: Later relationship conflict			
Social media use	.09	.03	[.03, .14]	.001
Time spent with colleagues				
Total effect	.04	.03	[-.03, .10]	.238
Direct effect	.04	.03	[-.02, .11]	.159
Indirect effect	-.005		[-.01, -.002]	
	Outcome: Later relationship conflict			
Social media use	.07	.03	[.13, .12]	.015
Boredom				
Total effect	.06	.02	[.03, .10]	.001
Direct effect	.05	.02	[.01, .09]	.006
Indirect effect	.01		[.002, .02]	
	Outcome: Later relationship satisfaction			
Relationship conflict	-.23	.01	[-.25, -.20]	<.001
Social media				
Total effect	-.05	.02	[-.09, -.01]	.006
Direct effect	-.03	.02	[-.06, .01]	.127
Indirect effect	-.02		[-.03, -.01]	

3) Analysis: within and between person analyses

First, we tested a model (with the same multilevel structure as in the original analyses) in which we examined the within person and between person effects cross-sectionally, including, similarly to the above analyses for Study 1, the within-person centered predictor variable as well as its aggregate, to disentangle the within- and between-person differences (Bolger & Laurenceau, 2013; Zhang et al., 2009).

As expected, in separate models, we regressed social media use on each pandemic factor and found that social media use was significantly associated at both a between and within-person level with all pandemic factors: pandemic seriousness (between person: b =.14, SE = .02, 95% CI [.10, .18], p <.001; within person: b = .17, SE = .01, 95% CI [.15, .20], p < .001), perception of personal threat (between person: b = .16, SE = .01, 95% CI [.13, .19], p < .001; within person: b = .10, SE = .01, 95% CI [.07, .12], p < .001), time spent with friends face to face (between person: b = -.08, SE =. 01, 95% CI [-.14, -.04], p < .001; within person: b = -.08, SE = .02, 95% CI [-.11, -.05], p < .001), time spent with family face to face (between person: b = .02, SE =. 01, 95% CI [.005, .05], p < .001; within person: b = .02, SE = .01, 95% CI [-.003, .04], p =.10), time spent with colleagues face to face (between person: b = -04, SE =. 02, 95% CI [-.08, -.001], p = .048; within person: b = -.10, SE = .02, 95% CI [-.14, -.06], p < .001), boredom (between person: b = .20, SE = .01, 95% CI [.18, .23], p < .001; within person: b = .07, SE = .01, 95% CI [.05, .09], p < .001). These results parallel those found in the original analyses and indicate that people who experienced greater pandemic stressors as well as greater fluctuations across the time periods (with the exception of less time spent with family, for which only between person effects were significant) also reported spending more time on social media.

Next, in separate models, we regressed relationship conflict on social media use (within person and aggregate), controlling for each pandemic factor (within person and aggregate). Results revealed a significant association between social media use and conflict, but only at the between person level. That is, people who spent more time on social media compared to others (but not necessarily those who experienced greater fluctuations in their social media use over time) reported greater conflict with their partner. Indirect effects for between-person values were calculated and did not contain zero, indicating that social media use was a significant mediator between pandemic factors and relationship conflict in all models, at the between person level.

Finally, to test the full serial mediation, we tested whether social media use was related to relationship satisfaction through the mediating role of conflict. First, we regressed relationship conflict on social media use and found a significant between person association (*b* = .19, *SE* = .03, 95% CI [.13, .26], p < .001) but not within person (*b* = -.01, *SE* = .03, 95% CI [-.07, .06], *p* = .797). Second, we regressed relationship satisfaction on conflict controlling for social media use, and found a significant association. Again, indirect effect did not contain zero, indicating that social media use was related to lower relationship satisfaction through greater conflict with the partner at the between person level. These results indicate that people who experienced greater pandemic stressors, also engaged in greater social media use and reported worse

relationship quality, but fluctuation in people's own social media use did not drive these effects. Full results can be seen in Table 4.

Table 4: Results of the cross-sectional multilevel mediation analysis in Study 2 on the effect of pandemic seriousness, pandemic threat, and time spent with friends, family, or colleagues, and boredom on relational outcomes through social media use and relationship conflict. * indicates *b* pathways in the mediation models and were estimated controlling for *a* pathways of the predictor variables.

	Between-person effects				Within-person effect			
Predictor and effect	*b*	*SE*	95% CI	*p*	*b*	*SE*	95% CI	*p*
				Outcome: Relationship conflict				
Social media use * Pandemic seriousness	.19	.03	[.12,.26]	<.001	-.01	.04	[-.08, .06]	.724
Total effect	.06	.04	[-.01, .13]	.100	.02	.02	[-.04, .07]	.609
Direct effect	.03	.04	[-.04, .11]	.361	.02	.03	[-.04, .07]	.566
Indirect effect	.03		[.02, .04]		-.002		[-.01, .01]	
				Outcome: Relationship conflict				
Social media use * Pandemic threat	.15	.03	[.08, .22]	<.001	-.01	.03	[-.08, .06]	.762
Total effect	.18	.03	[.13, .26]	<.001	.01	.02	[-.04, .06]	.670
Direct effect	.16	.03	[-.04, .06]	.641	.01	.02	[-.04, .06]	.641
Indirect effect	.02		[.01, .03]		-.001		[-.01, .01]	
				Outcome: Relationship conflict				
Social media use * Time spent with friends	.19	.03	[.13, .26]	<.001	-.01	.03	[-.08, .06]	.810
Total effect	.02	.04	[-.07, .10]	.670	.01	.03	[-.06, .07]	.821
Direct effect	.04	.04	[-.05, .12]	.400	.01	.03	[-.06, .07]	.838
Indirect effect	-.02		[-.02, -.01]		.001		[-.01, .01]	
				Outcome: Relationship conflict				
Social media use * Time spent with family	.19	.03	[.12, .26]	<.001	-.01	.03	[-.08, .06]	.818
Total effect	-.02	.02	[-.07, .02]	.305	.01	.02	[-.03, .06]	.573
Direct effect	.01	.02	[-.04, .05]	.740	-.02	.02	[-.07, .02]	.309
Indirect effect	.004		[.001, .008]		.0002		[-.002, .001]	
				Outcome: Relationship conflict				
Social media use * Time spent with colleagues	.20	.03	[.13, .26]	<.001	-.01	.03	[-.08, .05]	.720
Total effect	.04	.04	[-.04, .12]	.368	-.05	.04	[-.14, .03]	.232
Direct effect	.05	.04	[-.03, .13]	.263	-.05	.04	[-.14, .03]	.223
Indirect effect	-.01		[-.02, -.001]		.001		[-.01, .01]	
				Outcome: Relationship conflict				
Social media use *	.09	.04	[.02, .16]	<.001	-.02	.03	[-.09, .04]	.509

Boredom								
Total effect	.25	.02	[.21, .30]	<.001	.07	.02	[.03, .12]	.002
Direct effect	.24	.02	[.19, .28]	<.001	.07	.02	[.03, .12]	.002
Indirect effect	.02		[.002, .03]		-.002		[-.007, .003]	
Outcome: Relationship satisfaction								
Conflict * Social media use	-.35	.01	[-.38, -.32]	<.001	-.22	.01	[-.24, -.20]	<.001
Total effect	-.04	.03	[-.09, .02]	.194	.03	.02	[-.01, .08]	.140
Direct effect	.03	.03	[-.02, .08]	.192	.03	.02	[-.01, .07]	.147
Indirect effect	-.07		[-.09, -.05]		.002		[-.01, .02]	

4) Number and Percent of Participants Represented from each Country at Wave 1

Country	N	%
Australia	14	.5
Austria	3	.1
Azerbaijan	1	.0
Bahrain	1	.0
Belgium	8	.3
Brazil	6	.2
Canada	152	4.9
Central African Republic	4	.1
Chile	2	.1
China	90	2.9
Colombia	11	.4
Costa Rica	4	.1
Croatia	5	.2
Czech Republic	2	.1
Denmark	2	.1
Ecuador	1	.0
Finland	5	.2
France	30	1.0
Germany	22	.7
Greece	1	.0
India	1	.0
Indonesia	80	2.6
Ireland	2	.1
Italy	109	3.5
Japan	2	.1

Kazakhstan	2	.1
Luxembourg	1	.0
Macau	13	.4
Malaysia	2	.1
Mexico	13	.4
Netherlands	91	2.9
Nicaragua	3	.1
Niger	2	.1
Norway	2	.1
Peru	1	.0
Poland	4	.1
Portugal	157	5.0
Romania	4	.1
Russian Federation	1	.0
Saudi Arabia	1	.0
Singapore	2	.1
Slovakia	2	.1
Slovenia	1	.0
South Korea	6	.2
Spain	729	23.4
Sweden	8	.3
Switzerland	208	6.7
Taiwan	1	.0
Thailand	79	2.5
Turkey	242	7.8
United Kingdom	33	1.1
United States	944	30.4
Total	3110	100%

5) Power analyses

We conducted simulations for power analyses in R software (version 3.6.3) following the approaches recommended by Lane and Hennes (2018) and Kumle et al. (2020. We simulated 1,000 random data sets for Studies 1 and 2 capturing their structure. In each random sample the average correlations among predictors, percentage of missing data, and variables (predictors of technology use, social media use, relationship difficulties and relationship satisfaction) with fixed effect estimates, random intercept, and residual variance were set to be the same as in the original samples. We conducted three types of power analyses (post-hoc power analyses, analyses based on previous literature, and the SESOI method with analyses

estimating the smallest effect size of interest detectable in a replication), as described below.

First, power analyses for actual results were conducted post-hoc. Although this approach is no longer recommended, we include it here as a comparison to the other approaches that have been used for multilevel models. As Table 1 shows (approach 1), Study 1's data showed 76% and 82% power, respectively, to detect the associations between lockdown and self phone and partner phone use, and 72% power between phone use and relationship difficulties. The link between relationship difficulties and satisfaction has >.80% power. In Study 2, data showed .77 and >.80% power to detect the associations between social media use and relationship difficulties, and between relationship difficulties and satisfaction, respectively. Regarding social media use predictors, while pandemic serious, threat and boredom showed >. 80%, the connection between times spent with friends, family, and colleagues, showed between 42-100% power.

Second, we tested how much power the data sets for Studies 1 and 2 have to detect reasonable effects based on previous literature (approach 2). Unstandardized effects sizes from prior research appear in Table 1. Specifically, we used McDaniel and Drouin's (2019) effect to test the association between technology use and relationships difficulties, and McDaniel et al. (2020) for the relationship of difficulties and satisfaction, given that these works seem to represent the general effect sizes reported in the relevant literature. Given the recency of pandemic and its unique characteristics, it is more difficult to determine what a reasonable expected effect size would be for the lockdown → phone use pathway in Study 1. After careful consideration, we chose papers that examined internet and social media use before and after public health crises in China (Lyu, 2012) and before and during lockdown in Italy (Cellini et al., 2020), given that the context of crisis and the study designs in these papers largely reflected the conditions in our Study 1, with phone use assessed before and during lockdown. Results revealed that Study 1 had between 63% and 87.2% power to predict phone use. For Study 2, we used Stockdale and Coyne's (2020) effects of the motives for technology use (connection, information seeking and boredom) for our estimations, as they better mirrored the variables and design used in our study as predictors of social media use. Results revealed that pandemic seriousness, threat, boredom, and time spent with family, friends, and colleagues have >.80% power to predict social media use. Finally, both Studies 1 and 2, have >.80% power to detect the associations between technology use and relationship difficulties, and between relationship difficulties and satisfaction based on prior research effects. Thus, based on estimates from previous literature, our studies appear to generally have adequate power to detect the effects of interest.

Third, given that the prior two approaches to estimating power are subject to potential biases (e.g. even adopting effect sizes from published data involves the risk of performing the analyses on inflated effect sizes, Kumle et al., 2020), we also estimated power by progressively decreasing the estimates of the focal associations in our Studies until power was 80% (approach 3). This approach enabled us to identify the smallest effect sizes that could be reliably detected in a replication study with the same sample size and parameters as those used in this project. The results revealed that a replication of Study 1 would provide 80% power to detect an unstandardized association of .38 between predictor of technology use and phone use (1.23% more than the association observed in the present study), and between predictor of technology use and partner phone use (3% less than the association

observed in the present study), 81% power to detect an unstandardized association of .11 between phone use and relationship difficulties (1.22% more than the association observed in the present study), and 85% power to detect an unstandardized association of between relationship difficulties and satisfaction .10 (79.2% less than the association observed in the present study). For Study 2, a replication study would have a 85% power to detect an unstandardized association of .045 between predictor of technology use and social media use (71.9–43.75% less than the association observed in the present study; excluding the predictor time spent with family which need an 2.25 more), 81% power to detect an unstandardized association of .10 between social media use and relationship difficulties (1.1 % than the association observed in the present study), and 86% power to detect an unstandardized association of -.04 between relationship difficulties and satisfaction (85.2% less than the association observed in the present study). These results indicate that, overall, a replication using the same sample size and method would have adequate power (>.80%) to detect effect sizes similar (if not even smaller) than the ones found in our studies.

Table 5. Power analyses for Study 1 and 2 using three different approaches (Approach 1: post-hoc analyses; Approach 2: analyses based on previous literature; Approach 3: power to detect smallest effect in a replication). Effect sizes are unstandardized.

Predictor and effect	b	Power
STUDY 1		
Pandemic -> Phone Use		
Approach 1. Data: Self Phone Use	.31	.76
Approach 1. Data: Partner Phone Use	.39	.82
Approach 2. (Lyu, 2012)	.41	.87
Approach 2. (Cellini et al., 2020)	.28	.63
Approach 3. Smallest effect of interest	.38	.80
Phone Use -> Difficulties		
Approach 1. Data: Self and Partner Phone Use	.09	.72
Approach 2 (McDaniel & Drouin, 2019)	.26	.99
Approach 3. Smallest effect of interest	.11	.81
Difficulties -> Satisfaction		
Approach 1. Data	-.48	1
Approach 2 (McDaniel et al., 2020)	-.18	.97
Approach 3. Smallest effect of interest	-.10	.85
STUDY 2		
Predictors -> Social Media Use		
Approach 1. Data: Pademic Seriousness	.16	1
Approach 1. Data: Pademic Threat	.13	1
Approach 1. Data: Time spent with friends	-.08	.1
Approach 1. Data: Time spent with family	.02	.42
Approach 1. Data: Time spent with colleagues	-.08	1
Approach 1. Data: Boredom	.14	1
Approach 2. Connection (Stockdale & Coyne, 2020)	.05	.92
Approach 2. Information Seeking (Stockdale & Coyne, 2020)	.11	1
Approach 2. Boredom (Stockdale & Coyne, 2020)	.11	1
Approach 3. Smallest effect of interest: Predictors technology use	.045	.85
Social Media Use -> Difficulties		

Approach 1. Data	.09	.77
Approach 2 (McDaniel & Drouin, 2019)	.10	.81
Approach 3. Smallest effect of interest	.10	.81
Difficulties -> Satisfaction		
Approach 1. Data	-.27	1
Approach 2 (McDaniel et al., 2020)	-.18	1
Approach 3. Smallest effect of interest	-.04	.86
